# A GLP-1 Analog Liraglutide Reduces Intimal Hyperplasia After Coronary Stent Implantation *via* Regulation of Glycemic Variability and NLRP3 Inflammasome/IL-10 Signaling in Diabetic Swine

**DOI:** 10.3389/fphar.2020.00372

**Published:** 2020-03-26

**Authors:** Jinggang Xia, Qinxue Li, Yayun Liu, Quanxin Ren, Jinhuan Gao, Yi Tian, Jubo Li, Baojie Zhang, Haichen Sun, Shuang Liu

**Affiliations:** ^1^Department of Cardiology, Xuanwu Hospital, Capital Medical University, National Clinical Research Center for Geriatric Diseases, Beijing, China; ^2^Beijing Fangshan District Liangxiang Hospital, Beijing, China; ^3^Beijing Anzhen Hospital, Capital Medical University, Beijing, China; ^4^Department of Animal Experimental Center, Fuwai Hospital, National Center for Cardiovascular Disease, China Academy of Medical Sciences, Beijing, China; ^5^Surgical Laboratory, Xuanwu Hospital, Capital Medical University, Beijing, China

**Keywords:** liraglutide, intimal hyperplasia, glycemic variability, NOD-like receptor family pyrin domain containing 3, interleukin-10

## Abstract

**Objective:**

This study aimed to explore whether treatment with the glucagon-like peptide-1 (GLP-1) analog liraglutide reduces intimal hyperplasia after coronary stent implantation *via* regulation of glycemic variability, the NLRP3 inflammasome, and IL-10 in diabetic swine.

**Methods:**

Fifteen pigs were divided into a diabetes mellitus (DM) group (n = 6), a DM + liraglutide treatment group (L group) (n = 6) and a sham group (n = 3). A total of 24 everolimus-eluting stents were implanted in the left anterior descending and right coronary arteries at 3 weeks. A novel continuous glucose monitoring system (GMS) was used for 2 weeks. The means and standard deviations (SDs) were measured and calculated by the GMS. At 22 weeks, the lumen area (LA), neointimal thickness (NIT), neointimal area (NIA), and percent area stenosis (%AS) were analyzed by optical coherence tomography. Plasma tumor necrosis factor-*α*, interleukin-6, and interleukin-10 were assayed by ELISA. The intima protein expression levels of NLRP3, interleukin-1*β*, interleukin-18 and interleukin-10 were examined using Western blot analysis. Histology was used to evaluate the healing response. In an *in vitro* study, THP-1 cells were divided into control, high glucose (HG), HG + liraglutide, and HG + liraglutide + Exe(9–39) (a GLP-1 receptor inhibitor) groups.

**Results:**

The L group had a lower SD, NIT, NIA, and %AS; a larger LA; reduced inflammation and injury scores; lower expression levels of tumor necrosis factor-*α*, interleukin-6, NLRP3, interleukin-1*β*, and interleukin-18; and higher expression of interleukin-10 compared with those of the DM group (*p* < 0.05). In the *in vitro* study, similar results were obtained in the HG + liraglutide group, and Exe(9–39) abolished the effect of liraglutide (*p* < 0.05).

**Conclusions:**

Liraglutide treatment reduces intimal hyperplasia after stent implantation *via* regulation of glycemic variability, the NLRP3 inflammasome, and IL-10 in diabetic pigs in a GLP-1 receptor-dependent manner. Reducing the inflammation induced by glycemic variability may be one of the cardioprotective mechanisms of liraglutide.

## Introduction

Diabetes mellitus (DM) is continuously increasing globally and is associated with a wide spectrum of vascular complications, particularly coronary artery disease (CAD). There have been remarkable improvements in the outcomes of percutaneous coronary intervention (PCI) for the treatment of CAD with the use of second-generation everolimus-eluting stents (EES). Diabetic patients are a group of patients who are at an increasing risk of restenosis and target lesion revascularization. Antidiabetic and cardiovascular protective medicine is expected to improve the prognosis of coronary heart disease (Bruen et al., 2017; Bretón-Romero et al., 2018). In the LEADER trial, liraglutide treatment significantly reduced major cardiac events in diabetic patients who had a high risk of cardiovascular disease (Kim and Kim, 2017), which may be explained by the antiatherosclerotic and anti-inflammatory mechanisms of liraglutide (Hirano and Mori, 2016). However, the effects and possible mechanism of liraglutide on neointimal hyperplasia after coronary stent implantation remain elusive. Accumulating evidence suggests roles for NOD-like receptor family pyrin domain containing 3 (NLRP3) in mediating inflammation and interleukin-10 (IL-10) in mediating anti-inflammatory effects in diabetes and cardiovascular diseases (Groslambert and Py, 2018). The present study aimed to determine whether the glucagon-like peptide-1 (GLP-1) analog liraglutide reduces intimal hyperplasia after coronary stent implantation and inflammatory regulation in diabetic pigs.

## Materials and Methods

### In Vivo Study

#### Animals

The preclinical study protocols followed the Guide for the Care and Use of Laboratory Animals published in 1996 by the US National Institutes of Health. The protocols were approved by the Ethics Committee of Xuanwu Hospital of Capital Medical University and were performed in accordance with the Declaration of Helsinki.

**Figure 1:**
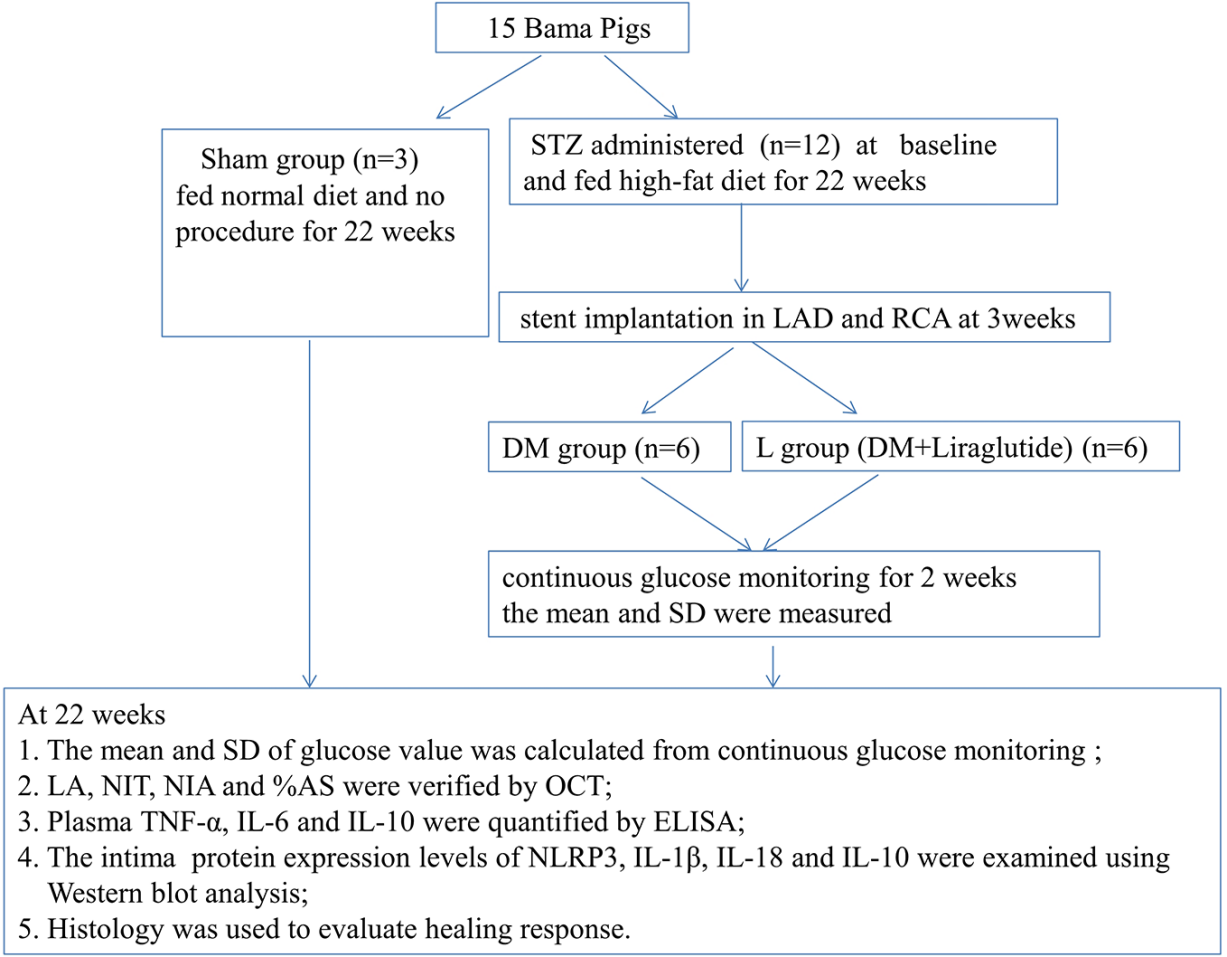
Study protocol. DM, diabetes mellitus; STZ, streptozotocin; RCA, right coronary artery; LAD, left anterior descending; SD, standard deviation; LA, Lumen area; NIT, neointimal thickness; NIA, neointimal area; %AS, percent area stenosis. OCT, optical coherence tomography; TNF-*α*, tumor necrosis factor-*α*; IL-6, interleukin-6; IL-10, interleukin-10; NLRP3, NOD-like receptor family pyrin domain containing 3; IL-1*β*, interleukin-1*β*; IL-18, interleukin-18.

The flow chart of the study is shown in **Figure 1**. Fifteen Bama pigs (male, aged 5 months, weighing 35–40 kg) were obtained from the breeding factory of China Agricultural University. They were divided into a DM group (n = 6), a DM + liraglutide treatment group (L group, n = 6), and a sham group (n = 3) using a random number table. The pigs in the DM and L groups were each administered a single dose of streptozotocin (STZ) (100 mg/kg body weight, Sigma 0130, St. Louis, Missouri, USA) intravenously at baseline, followed by a high-fat diet (2% cholesterol and 20% lard) for 22 weeks. A consistent blood glucose level >150 mg/dl was considered to be successful in establishing a diabetes model. Intermediate-acting and short-acting insulin therapies were initiated, if necessary, to maintain glucose levels below 350 mg/dl to prevent ketoacidosis. The sham group was fed a normal diet and did not receive intravenous STZ injections or the stent implantation procedure. The pigs were fed at a level of 2.5% of their body weight daily. All pigs were housed and monitored with veterinary care at the animal department of Capital Medical University.

#### Stent Implantation and Liraglutide Treatment

At the beginning of 3 weeks, 24 coronary sites for stent implantation were selected after angiography in 12 diabetic pigs; the sites were located in the proximal to midsection of the left anterior descending (LAD) and right coronary arteries (RCA). A total of 24 EESs (Xience Xpedition, Abbott, USA) were implanted, targeting up to 20% overstretch. All pigs were observed until 22 weeks. Angiography and optical coherence tomography (OCT) were performed at the end of 22 weeks. The six pigs in the L group received subcutaneous injections of liraglutide (0.005 mg/kg/day) referring to the previous publication (Sassoon et al., 2017) after stent implantation at 3 weeks until the end of 22 weeks.

#### Experimental Procedures

The 12 diabetic pigs were pretreated with dual antiplatelet therapy (300 mg clopidogrel and 300 mg aspirin) 12 h prior to the surgery. After anesthetization, the pigs were connected to the mechanical ventilator through endotracheal intubation. Arterial femoral access was then obtained *via* the Seldinger technique. The pigs were intravenously administered 5,000–10,000 U heparin to maintain an activated clotting time between 250 s and 300 s. Following coronary angiography, quantitative coronary analysis was performed before and after stent implantation. After successful coronary stent implantation, the pigs were extubated and then returned to the animal care facilities. At 22 weeks, the pigs were anesthetized, and the outcomes of stent implantation were evaluated by angiography and OCT.

#### Installation of the Glucose Monitoring System

FreeStyle Libre H (Abbott Diabetes Care, Alameda, CA) flash GMS was used to monitor interstitial glucose *via* subcutaneous sensor filaments that were adhered onto the internal femoral skin of pigs in the DM and L groups after stent implantation at 3 weeks. The GMS automatically measured glucose every minute up to 2 weeks (at 4‒5 weeks). The sensor collected glucose measurements and trends every 15 minutes and stored up to 8 h of glucose readings (Ji et al., 2017). The estimated glycated hemoglobin (HbA1c), the means, and standard deviations (SDs) based on the blood glucose values were measured and calculated automatically by the GMS.

#### OCT Imaging Protocol and Analysis

OCT was performed by operators with a C7-XROCT imaging system (Light-Lab Imaging, Inc., St. Paul, MN, USA) using nonocclusive techniques, which allowed the creation of a blood-free environment for imaging by the flushing of contrast media with a guiding catheter. OCT images were acquired with a motorized automatic pullback system at a speed of 20 mm/s and an acquisition rate of 100 frames per second. Three cross-sectional images were selected per pullback, the proximal stent, midstent, and distal stent. The images were analyzed in a LightLab Imaging System (Light Lab Imaging, Inc., Massachusetts, Westford, MA, USA). The lumen area (LA) and stent area (SA) were measured on each image to calculate the neointimal area (NIA) by using the formula LA − SA, as well as percent area stenosis (%AS) by using the formula [1 − (LA/SA)] × 100. To analyze neointimal thickness (NIT), the distance between the center of each stent strut and the luminal border was determined in the direction of the center of gravity.

#### Blood Samples

Blood samples were obtained at baseline and 22 weeks. Blood samples were obtained at any time if the pig was in poor condition. Fasting blood glucose (FBG), high-density lipoprotein cholesterol (HDL-C), low-density lipoprotein cholesterol (LDL-C), total cholesterol (TC), and triglyceride (TG) levels were determined using routine methods. Plasma interleukin-6 (IL-6), interleukin-10 (IL-10), and tumor necrosis factor-alpha (TNF-*α*) were assayed by using commercially available ELISA kits (Santa Cruz, USA) according to the manufacturer's instructions.

#### Histology and Western Blot Analysis of Related Markers

At 22 weeks after the OCT procedure, a thoracotomy was performed. The heart from each pig was excised. The coronaries were properly perfused with 0.9% saline through the ascending aorta to remove the blood and then fixed with 10% buffered formalin for 1 h or longer. The stented arterial segments were then excised, fixed with formalin overnight, dehydrated with graded ethanol solutions and embedded in methyl methacrylate resin. Proximal, mid, and distal parts of three cross-sections (4–6 μm) were obtained from each vessel on a rotary microtome and stained with hematoxylin and eosin (HE) and Masson staining.

The cross-sections were evaluated for inflammation score (0–4), fibrin score (0–3), and vessel injury score (Schwartz) (0–3) by using a semiquantitative method as previously described (**Supplementary Table 1**) (Takimura et al., 2015; Sperling et al., 2019).

The intima of the stent segment edge was also collected, and total protein was extracted as previously described (Xia et al., 2014). The membranes were incubated with primary antibodies overnight at 4°C as follows: NLRP3 (1:1,000; Novus, USA), interleukin-1*β* (IL-1*β*; 1:1,000; Novus, USA), interleukin-18 (IL-18; 1:1,000; Novus, USA), IL-10 (1:1,000; R&D, USA), and *β*-actin (1:2,000; Anhui, China). Then, the membranes were subsequently incubated with their corresponding secondary antibody at room temperature for 1 h. The blots were developed by using enhanced chemiluminescence (Millipore, Billerica, MA, USA). The immunoreactive results were analyzed with ImagePro Plus 6.0 software and corrected by normalization to the value of *β*-actin.

### *In Vitro* Studies

THP-1 cells were obtained from the Cell Bank of the Chinese Academy of Sciences in China and were cultured in RPMI 1640 medium (containing HEPES; Gibco, USA) supplemented with 10% fetal bovine serum (Biological Industries, USA) and 0.05 mM *β*-mercaptoethanol (Sigma-Aldrich, USA) and streptomycin (100 μg/ml) in an incubator with 5% CO_2_ at 37°C. PMA (160 nM phorbol-12-myristate acetate; Sigma-Aldrich, USA) was used to stimulate THP-1 monocytes at a density of 1 × 10^6^ cells/ml for 3 days to induce their differentiation into macrophages.

THP-1 cells were then divided into the following four groups: (1) control group (C group), which was cultured in RPMI 1640 medium for 6 h; (2) high glucose (HG) group, which was cultured in RPMI 1640 medium plus 25 mmol/L D-glucose for 6 h; (3) HG + liraglutide (L) group, which was pretreated with 100 nmol/L liraglutide 3 h prior to 25 mmol/L D-glucose treatment in RPMI 1640 medium for 6 h; and (4) HG +L + Exe(9–39) group, which was pretreated with exe(9–39) 200 nmol/L 3 h prior to 100 nmol/L liraglutide treatment for 3 h, followed by 25 mmol/L D-glucose in RPMI 1640 medium for 6 h. The protein expression of NLRP3 and IL-10 was evaluated in the four groups by Western blotting.

### Statistical Analysis

Statistical analyses were performed using SPSS 13.0. The data are expressed as the mean ± SD unless otherwise specified. The difference between two variables was analyzed by Student's t-test. One-way analysis of variance (ANOVA) was used to examine differences among multiple comparisons. Percentages were compared using the chi-squared test or Fisher's exact test where appropriate. A two-sided *p* value of < 0.05 was considered statistically significant.

## Results

### Body Weights and Blood Samples

At 22 weeks, body weight significantly decreased in the DM and L groups compared with that of the sham group (*p* < 0.05). Body weight was similar between the DM and L groups (*p* > 0.05). FBG, TC, LDL-C, TG, IL-6, IL-10 and TNF-*α* were significantly increased in the DM and L groups compared with those of the sham group (*p* < 0.05). HDL-C significantly decreased in the DM and L groups compared with that of the sham group (*p* < 0.05). FBG, TC, LDL-C, HDL-C and TG were similar between the DM and L groups (*p* > 0.05). TNF-*α* and IL-6 significantly decreased in the L group compared with those of the DM group (*p* < 0.05). IL-10 significantly increased in the L group compared with that of the DM group (*p* < 0.05) (**Table 1**).

**Table 1:** Comparison of body weight and blood samples.

Parameter	at baseline			*P* Value	at 22 weeks			*P* Value
	Sham	DM	L group		Sham	DM	L group	
Body weight (kg)	35 ± 2.5	34 ± 2.9	36 ± 2.7	0.66	37 ± 4.6	31 ± 2.3^*^	31 ± 2.8^*^	0.03
Fasting blood glucose (mmol/L)	4.3 ± 1.2	4.2 ± 1.6	4.21 ± 1.8	0.88	4.8 ± 1.5	11.8 ± 2.3^*^	10.2 ± 2.6^*^	0.01
Total cholesterol (mmol/L)	2.6 ± 0.5	2.7 ± 0.6	2.6 ± 0.8	0.91	2.8 ± 1.7	5.5 ± 0.8^*^	5.1 ± 0.6^*^	0.02
Low-density lipoprotein cholesterol (mmol/L)	1.45 ± 0.4	1.43 ± 0.5	1.46 ± 0.7	0.89	1.72 ± 0.8	4.7 ± 1.1^*^	4.5 ± 0.9^*^	0.001
High-density lipoprotein cholesterol (mmol/L)	1.02 ± 0.19	1.11 ± 0.21	1.08 ± 0.21	0.55	1.05 ± 0.21	0.75 ± 0.15^*^	0.79 ± 0.11^*^	0.04
Triglycerides (mmol/L)	0.5 ± 0.1	0.6 ± 0.3	0.5 ± 0.3	0.92	0.9 ± 0.5	2.2 ± 0.7^*^	2.1 ± 0.6^*^	0.01
Tumor necrosis factor-alpha (pg/ml)	205 ± 15	210 ± 19	208 ± 21	0.73	212 ± 17	350 ± 26^*^	255 ± 23*^#^	0.01
Interleukin-6 (pg/ml)	187 ± 12	193 ± 21	197 ± 19	0.69	196 ± 13	365 ± 29^*^	298 ± 26^*#^	0.003
Interleukin-10 (pg/ml)	192 ± 16	189 ± 22	179 ± 25	0.75	202 ± 15	259 ± 22^*^	357 ± 28^*#^	0.001

DM, diabetes mellitus; L, DM+ liraglutide treatment.

*indicates a significant difference compared to sham, ^#^indicates a significant difference compared to DM group.

**Figure 2:**
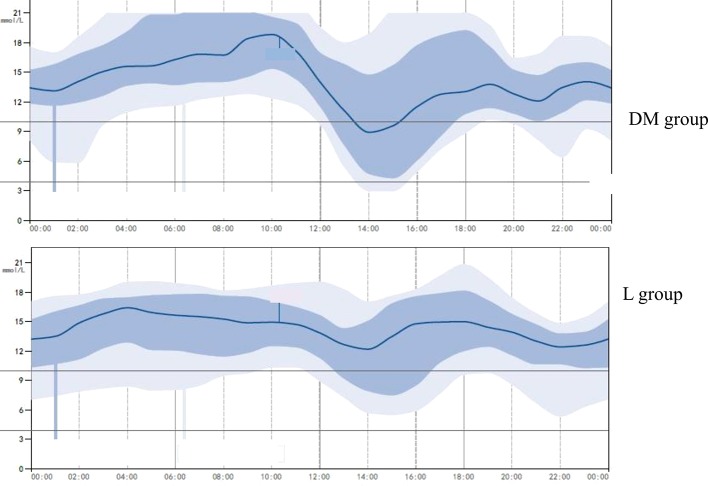
A representative glucose-monitoring showing higher glycemic variability in diabetes mellitus group and lower glycemic variability in liraglutide treatment group. L group, liraglutide treatment group.

No significant difference was found in the estimated HbA1c between the DM and L groups (10.7 ± 1.8% *vs.* 10.2 ± 2.1% *p* > 0.05). No significant difference was found in the mean blood glucose between the DM and L groups (13.2 ± 1.4 mmol/L *vs.* 12.8 ± 1.5 mmol/L *p* > 0.05), and the SD of the blood glucose was significantly lower in the L group compared to that of the DM group (2.59 ± 0.21 mmol/L *vs.* 3.28 ± 0.31 mmol/L, *p* < 0.05), as measured by the GMS (**Figure 2**).

### OCT Analysis

**Figure 3:**
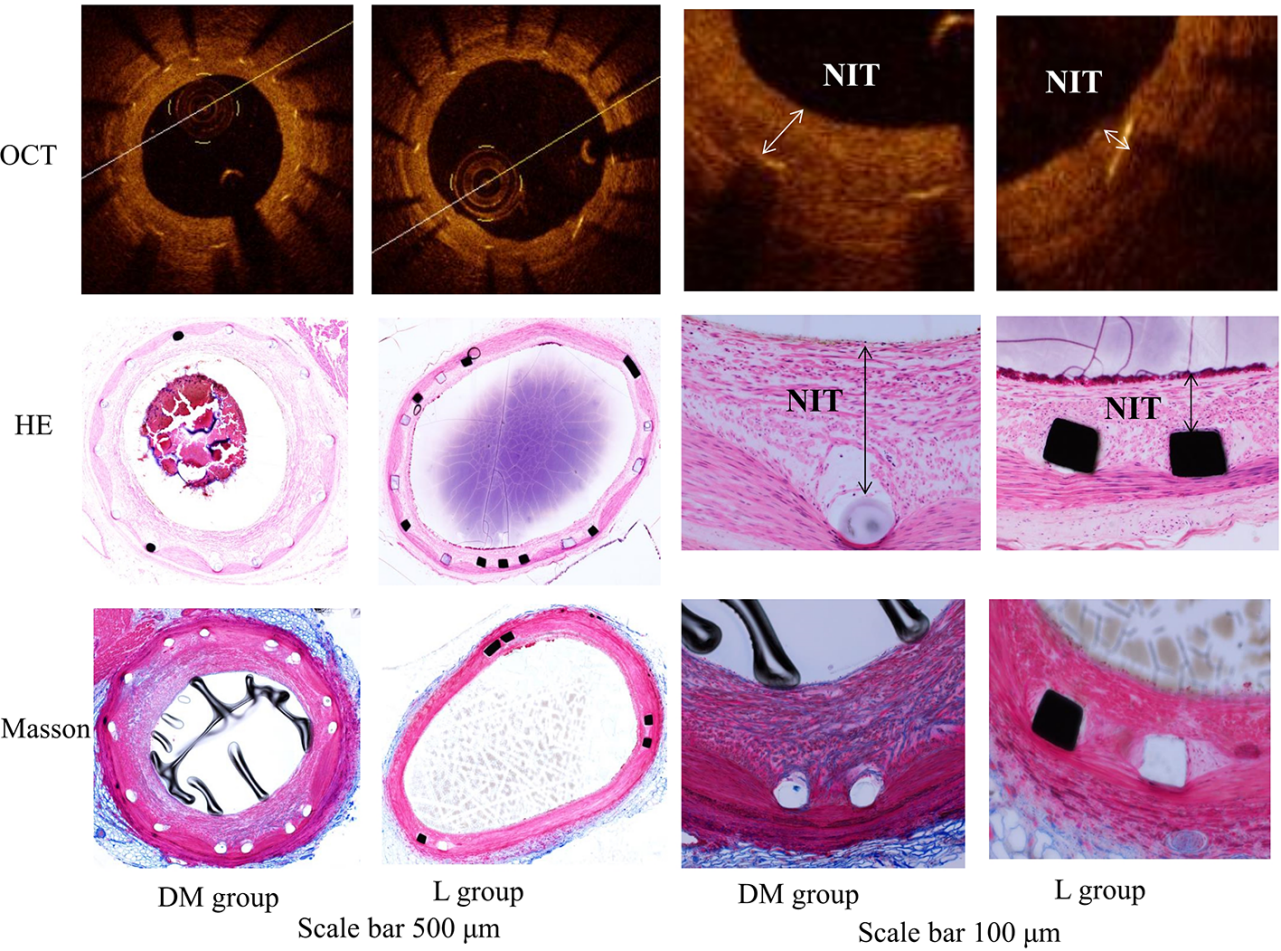
OCT image and histology of the representative vessels with HE and Masson staining in diabetes mellitus group and liraglutide treatment group. DM, diabetes mellitus; L group, liraglutide treatment group; OCT, optical coherence tomography. The liraglutide treatment group was associated with a larger lumen area and lower neointimal thickness, neointimal area and percent area stenosis compared with the DM group. The scale bar of two columns on the left is 500 μm and the scale bar of two columns on the right is 100 μm. NIT, neointimal thickness.

OCT analysis at 22 weeks is presented in **Table 2**. The SA was not significantly different between the DM and L groups. The L group was associated with a larger LA and lower NIT, NIA, and %AS compared with those of the DM group (*p* < 0.05). The representative vessels with OCT are presented in **Figure 3**.

**Table 2:** Summary of OCT in all treated vessels and semiquantitative histologic analysis at 22 weeks follow-up.

	DM group (n = 6)	L group (n = 6)	*p*
OCT analysis			
Lumen area (mm^2^)	2.65 ± 0.21	4.14 ± 0.65	<0.001
Stent area (mm^2^)	4.96 ± 0.85	5.22 ± 0.74	0.585
Neointimal area (mm^2^)	2.29 ± 0.31	1.08 ± 0.13	<0.001
Percent area stenosis (%)	44.31 ± 5.6	27.3 ± 3.7	<0.001
NIT(mm)	0.39 ± 0.09	0.25 ± 0.03	<0.01
Semiquantitative histologic analysis			
Inflammation score (0–4)	0.85 ± 0.32	0.47 ± 0.18	0.03
Fibrin score (0–3)	0.68 ± 0.29	0.75 ± 0.36	0.72
Injury score (0–3)	0.78 ± 0.27	0.42 ± 0.15	0.02

OCT, optical coherence tomography; DM, diabetes mellitus; L, DM + liraglutide treatment; NIT, neointimal thickness.

### Semiquantitative Histological Analysis

The L group showed lower inflammation and injury scores compared to those of the DM group (*p* < 0.05). The fibrin score was similar between the DM and L groups (*p* > 0.05). The semiquantitative histological analysis is presented in **Table 2**. Histology of the representative vessels with HE and Masson staining is presented in **Figure 3**.

### Western Blot Analysis of Intima Protein Expression Levels of the NLRP3 Inflammasome and IL-10

**Figure 4:**
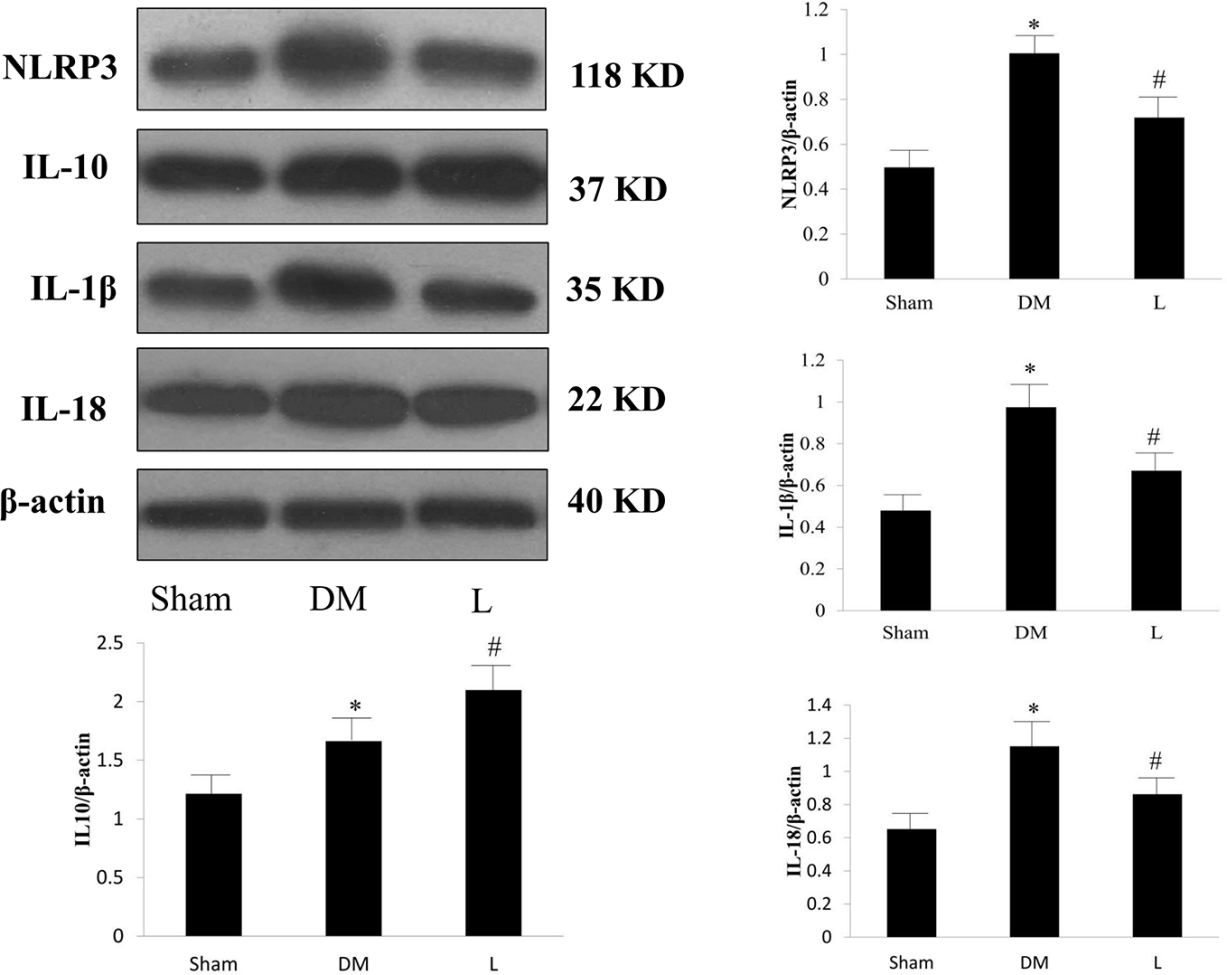
Protein expression levels of NLRP3, IL-1*β*, IL-18 and IL-10 in the Sham, DM, and liraglutide treatment groups. L, liraglutide treatment group; NLRP3, NOD-like receptor family pyrin domain containing 3; IL, interleukin; DM, diabetes mellitus. Comparisons among the three groups (n = 3 each group). Data are expressed as the mean ± SD. *p < 0.05 *vs.* sham group. ^#^*p* < 0.05 *vs.* DM group. Representative images of NLRP3, IL-1*β*, IL-18, and IL-10. *β*-actin serves as a reference.

In the *in vivo* study, the DM group exhibited significantly increased protein expression levels of NLRP3, IL-1*β*, IL-18, and IL-10 compared with those of the sham group (*p* < 0.05, **Figure 4**). Liraglutide treatment significantly abrogated the DM-induced increase in NLRP3, IL-1*β*, and IL-18 protein expression levels (*p <*0.05). Liraglutide treatment significantly increased IL-10 protein expression compared with that of the DM group (*p* < 0.05).

**Figure 5:**
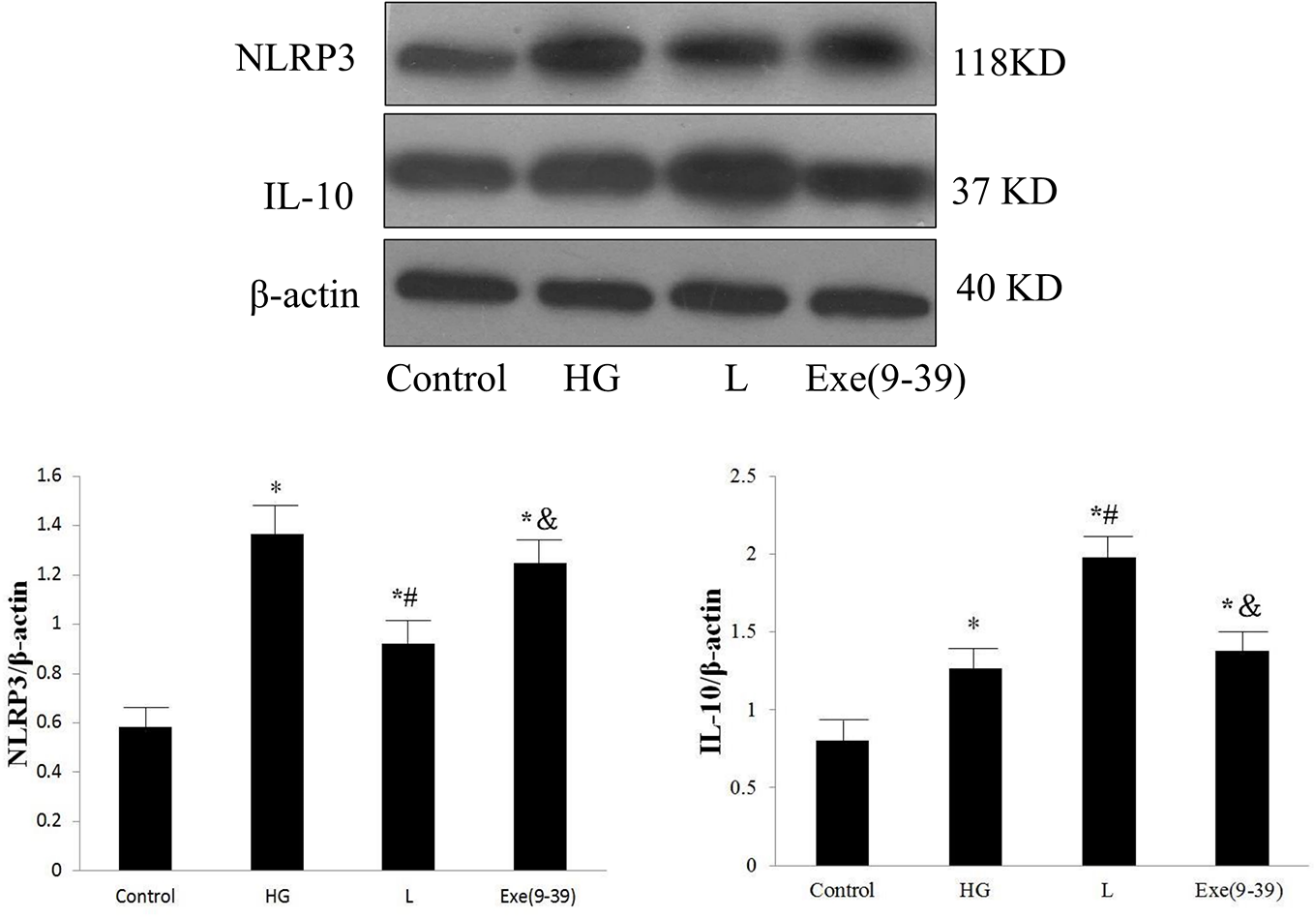
Protein expression levels of NLRP3 and IL-10 in the control group, HG group, HG + liraglutide group, and HG + liraglutide + Exe(9–39) group. HG, high glucose; L, HG + liraglutide treatment; Exe(9–39), HG + L + Exe(9–39) group; NLRP3, NOD-like receptor family pyrin domain containing 3; IL-10, interleukin-10. Comparisons among the four groups (n = 3 each group). The data are expressed as the mean ± SD. *p < 0.05 *vs.* the control group, ^#^*p* < 0.05 *vs.* the HG group, and *p* < 0.05 *vs.* the L group. Representative images of NLRP3 and IL-10. *β*-actin served as a reference.

In the *in vitro* study, the protein expression levels of NLRP3 and IL-10 in the HG group were significantly higher than those in the control group (*p* < 0.05). The L group was associated with reduced NLRP3 protein expression and further increased IL-10 protein expression compared with those of the HG group (*p* < 0.05). Exe(9–39) abolished the effect of liraglutide (*p* < 0.05). Representative Western blot images are shown in **Figure 5**.

## Discussion

In the current study, we explored whether the glucagon-like peptide-1 analog liraglutide reduces intimal hyperplasia after coronary stent implantation and the underlying mechanisms. We found that (1) liraglutide treatment was associated with increased LA and reduced NIT, NIA, and %AS compared with those of the DM group; (2) liraglutide treatment was associated with reduced glycemic variability (SD); (3) liraglutide treatment was associated with reduced inflammation and injury scores in semiquantitative stented vascular histological analysis, reduced expression levels of NLRP3, IL-1*β*, IL-18, TNF-*α*, IL-6 and increased expression of IL-10; and (4) the beneficial effect of liraglutide in regulating the NLRP3 inflammasome and IL-10 was dependent on the GLP-1 receptor. Our report is the first study to show the effects of the GLP-1 analog liraglutide on neointima hyperplasia after coronary stent implantation in preclinical diabetic pigs and its relationship with glycemic variability and inflammatory regulation.

In this study, we found that liraglutide treatment was associated with decreased intimal hyperplasia after coronary stent implantation compared with that of the DM group. This protective effect of liraglutide on the vascular intima is supported by a previous study. The study by Rizzo and colleagues (Rizzo et al., 2014) showed that liraglutide treatment for 8 months significantly decreased carotid intima-media thickness (IMT), and this beneficial effect of liraglutide was independent of glycemic or lipid levels. They also found that liraglutide treatment significantly reduced waist circumference and body mass index, facilitated glycemic and lipid control, and decreased carotid IMT during a follow-up of 18 months. Metabolic syndrome prevalence was significantly reduced (Rizzo et al., 2016). Dr. Li found that liraglutide attenuated the atherosclerotic lesion and plaque progression in aortic tissues induced by high fat diets followed by low-dose streptozotocin injection in type 2 DM rats (Li et al., 2017). Rizzo et al. proposed that liraglutide exerted favorable cardiovascular effects through its direct antiatherosclerotic action, as well as its ability to prevent plaque formation and progression beyond glycemic control (Rizzo et al., 2018). We also explored the cardioprotective mechanism of liraglutide after stent implantation. We found that liraglutide treatment was associated with reduced SD, and no significant difference was found in the mean blood glucose between the DM and L groups, as determined by the GMS. There are three important indicators for diabetes management, FBG, postprandial blood glucose and HbA1c. However, HbA1c might not adequately reflect deviations from the mean blood glucose level. Diabetic patients with identical HbA1c values can show significantly different daily glucose profiles in terms of frequency, amplitude and duration of glucose excursions. Thus, glycemic variability, as a variable that reflects the dynamic process of diabetes control, has received increasing attention. Evidence has shown a more deleterious effect of glycemic variability on coronary arteries compared with that of chronic, sustained hyperglycemia (Kuroda et al., 2015; Škrha et al., 2016; Valensi et al., 2017). The SD of blood glucose is another widely used parameter for the evaluation of intraday glycemic variability and has multiple advantages, including easy access, wide coverage and no application limits (DeVries, 2013; Inzucchi et al., 2015). Current studies have suggested that higher glycemic variability is associated with greater oxidative stress and more inflammation (Monnier et al., 2006; Wang et al., 2015). One of the important findings of our study is that liraglutide reduced glycemic variability. Liraglutide treatment may decrease glycemic variability-associated oxidative stress.

We also found that liraglutide treatment was associated with reduced inflammation and injury scores in semiquantitative histological analysis, reduced expression levels of NLRP3, IL-1*β*, IL-6, IL-18, and TNF-*α*, and increased expression of IL-10. Our study suggests that liraglutide plays a protective role by reducing not only systemic but also local stented vascular inflammation. Inflammation with the accumulation of macrophages plays a pivotal role in atherosclerosis. The NLRP3 inflammasome is an essential mediator that regulates the activation of caspase-1 and subsequent processing of pro-IL-1*β*, followed by IL-1*β*, and IL-18. The NLRP3 inflammasome induces vascular inflammation, leading to the progression of atherosclerosis (Karasawa and Takahashi, 2017; Lee et al., 2017). IL-10 is a potent anti-inflammatory cytokine that is released by activated immune cells. Recent evidence has shown that IL-10 inhibits inflammasome activation, which is independent of any effect on NLRP3 transcription (Ip et al., 2017). IL-10 inhibits the production of IL-1β *via* two known mechanisms. First, IL-10 overexpression results in a reduction in NLRP3 inflammasome activity, which suppresses caspase-1-dependent IL-1*β* maturation. Second, autocrine IL-10 promotes activation and transduction of transcription-3, reducing the abundance of pro-IL-1β (Sun et al., 2019). Our study showed that liraglutide reduced intimal hyperplasia *via* downregulation of the NLRP3 inflammasome and upregulation of IL-10. These observations were supported by previous studies (Rakipovski et al., 2018; Zhu et al., 2018; Sharma et al., 2018; Bruen et al., 2019). The protective effect of the GLP-1 receptor agonist liraglutide on blood vessels occurs *via* GLP-1 receptor-dependent or independent signals (Zhong et al., 2019). In the *in vitro* study, liraglutide treatment significantly decreased NLRP3 and increased IL-10 protein expression compared with that of the HG group. Exe(9–39) abolished the effect of liraglutide. Our results indicated that the beneficial effect of liraglutide on intimal hyperplasia *via* regulation of the NLRP3 inflammasome and IL-10 is dependent on the GLP-1 receptor.

This study has several limitations. First, we established an animal model similar to type-1 DM, while the most common type in humans is type 2 DM. Second, only a small sample was included to investigate the impact of liraglutide on intimal proliferation. Third, more research on positive and negative feedback *in vitro* signaling pathways might help clarify the mechanism.

## Conclusions

The present study showed that liraglutide treatment reduced intimal hyperplasia after stent implantation compared with that of the DM group. Liraglutide treatment correlated with reduced glycemic variability, decreased expression of the NLRP3 inflammasome and increased expression of IL-10 in a GLP-1 receptor-dependent manner.

## Data Availability Statement

The raw data supporting the conclusions of this article will be made available by the authors, without undue reservation, to any qualified researcher.

## Ethics Statement

The animal study was reviewed and approved by the Ethics Committee of Xuanwu Hospital of Capital Medical University.

## Author Contributions

Conceptualization: JX. Investigation: JX, QL, YL, and QR. Methodology: JG, YT, JL, BZ, HS, and SL. Writing original draft: JX, QL, YL, and QR. Writing–review and editing: JX.

## Funding

This research was supported by National Natural Science Foundation of China (No. 81770344), National Clinical Research Center for Geriatric Diseases, Beijing Key Clinical Speciality Development Project and the China young and middle-aged clinical research -VG fund (No. 2017-CCA-VG-043).

## Conflict of Interest

The authors declare that the research was conducted in the absence of any commercial or financial relationships that could be construed as a potential conflict of interest.
