# Supplementary material for: A GLP-1 Analog Liraglutide Reduces Intimal Hyperplasia After Coronary Stent Implantation via Regulation of Glycemic Variability and NLRP3 Inflammasome/IL-10 Signaling in Diabetic Swine
Source: Front Pharmacol. 2020 Mar 26;11:372. doi: 10.3389/fphar.2020.00372 (PMC7113385; doi:10.3389/fphar.2020.00372)
Supplement: Supplementary file 1 [file Table_1.docx]

**Supplementary Table 1** Definitions of inflammation score, fibrin score and vessel injury score

|  | **Scores** | **Description** |
| --- | --- | --- |
| **Inflammation score** |  |  |
|  | 0 | <25% struts, ≤10 inflammatory cells |
|  | 1 | <25% struts, >10 inflammatory cells |
|  | 2 | 25–50% struts, >10 inflammatory cells |
|  | 3 | >50% struts, >10 inflammatory cells |
|  | 4 | 2 or more struts, granulomatous inflammatory reactions |
| Fibrin score |  |  |
|  | 0 | no fibrin deposition |
|  | 1 | focal residual fibrin at any portion of the artery  or moderate fibrin deposition, <25% of arterial circumference |
|  | 2 | moderate fibrin involving, >25% of arterial circumference  or heavy fibrin deposition, <25% of arterial circumference |
|  | 3 | heavy fibrin deposition, >25% of arterial circumference |
| Injury score  (Schwartz) |  |  |
|  | 0 | IEL intact, media compressed but not lacerated |
|  | 1 | IEL lacerated, media typically compressed but not lacerated |
|  | 2 | internal elastic lacerated, media visibly lacerated; EEL intact but compressed |
|  | 3 | EEL lacerated, typically large lacerations of media extending through the EEL, stent struts sometimes residing in adventitia |

IEL, internal elastic laminal; EEL, external elastic lamina.
